# The association between attention deficit hyperactivity disorder (ADHD) and smoking experience or exposure to environmental tobacco smoke among children and adolescents

**DOI:** 10.18332/tid/157209

**Published:** 2023-01-30

**Authors:** Sunho Lee, Wanhyung Lee

**Affiliations:** 1 Department of Pediatrics, Gyeongsang National University Changwon Hospital, Changwon, Republic of Korea; 2 Department of Occupational and Environmental Medicine, Gil Medical Center, Gachon University College of Medicine, Incheon, Republic of Korea

**Keywords:** ADHD, smoking, diagnosis, adolescence, tobacco exposure

## Abstract

**INTRODUCTION:**

Direct and indirect smoking exposure is highly related to mental health in children. This study aimed to identify the association between exposure to smoking or secondhand smoke (SHS) and attention deficit hyperactivity disorder (ADHD).

**METHODS:**

We used data from the Korean National Health and Nutrition Examination Survey (KNHANES) from 2007 to 2019, including variables such as diagnosed ADHD, smoking status, SHS, and urine cotinine levels among children and adolescents. We estimated the risk of ADHD according to smoking or SHS exposure in various exposure groups using adjusted logistic or linear regression models.

**RESULTS:**

Among 16434 participants, 133 children were diagnosed with ADHD (0.8%). Of these, 58 (43.6%) were aged <12 years and 75 (56.3%) were aged ≥12 years. Smoking was significantly associated with ADHD (crude odds ratio, OR=1.48; 95% CI: 1.14–3.26 and adjusted odds ratio, AOR=1.22; 95% CI: 1.02– 1.64). SHS exposure and ADHD were attenuated after adjustment (OR=2.42; 95% CI: 1.08–4.02; AOR=1.42; 95% CI: 0.86–2.64) in the logistic regression model. Smoking history was statistically associated with a younger age of ADHD diagnosis in the linear regression model.

**CONCLUSIONS:**

Smoking and the amount of smoking among children and adolescents was associated with ADHD.

## INTRODUCTION

Attention deficit hyperactivity disorder (ADHD) is a neurobehavioral disorder characterized by hyperactivity, impulsivity, and inattention in children and adolescents^1^. The Diagnostic and Statistical Manual of Mental Disorders (DSM) criteria are used to diagnose ADHD based on the symptoms and rating performance for diagnosis acquired by the teacher and parents or through a self-questionnaire. The overall prevalence of ADHD is 3–8% and is dependent on different regions: 6.2% in North America, 4.7% in Europe, 3.7% in Asia, and 2.3% in the Middle East^2^. In Korea, the prevalence of ADHD was reported variously by data or the type of study^3,4^. A current report indicated the rate of diagnosed ADHD treated with medications was 0.8% in South Korea^5^. Additionally, the prevalence of each type of ADHD differed^6^. Therefore, aberrant prevalence rates indicate various factors that influence ADHD.

Most studies analyzing the risk factors for ADHD assessed genetic inheritance and biological or environmental factors^7^. Among the various factors, smoking was highly associated with the health burden of ADHD in children and adolescents^8,9^. The longitudinal effect of tobacco smoking may affect frontal cortical thickness^10^. Structural MRI studies of ADHD have revealed volume reduction of cortical thickness abnormalities in the frontal and parieto-temporal regions^11^. An animal model randomly exposed to tobacco smoke had neuronal cell damage and a decreased brain surface^12^. However, the question of whether smoking influences ADHD remains.

Prenatal smoking increases the risk of ADHD^13,14^. The United Kingdom prospective cohort study revealed the association between maternal smoking during pregnancy and child ADHD^15^, and the Danish National Birth Cohort indicated that prenatal nicotine exposure might have a causal role in ADHD^16^. A Finnish nationwide report revealed a dose-response relationship between nicotine exposure during pregnancy and offspring ADHD^17^. However, the Norwegian mother and child cohort study suggested that prenatal maternal smoking is not firmly related to offspring ADHD compared to paternal and grandmother smoking^18^.

Exposure to environmental tobacco smoke (SHS) is associated with a health burden on children’s organ systems^19^. The National Survey on Children’s Health in the US found that secondhand smoke exposure is associated with childhood neurobehavioral disorders, including ADHD^20^. The Korean version of the study of ADHD rating scales reported that SHS in children is associated with a decline in neurocognitive performance^21^. Nevertheless, the association between SHS exposure and ADHD remains unclear^22^. Thus, the causality of these inverse associations needs, is clarified.

Using data from the Korean National Health and Nutrition Examination Survey (KNHANES) from 2007 to 2019, we investigated the risk of related factors of ADHD among Korean children and adolescents, focusing on smoking status and SHS exposure.

## METHODS

### Data and study participants

The Korea National Health and Nutrition Examination Survey (KNHANES) is a series of nationally representative, cross-sectional, population-based surveys of Koreans’ health and nutritional status conducted by the Korea Centers for Disease Control and Prevention. This annual survey uses a stratified and multistage probability sampling design to select household units, including non-institutionalized Korean civilians. Approximately 200 geographical sampling units from 17 central provinces of the Republic of Korea were used in the KNHANES 2007–2019. In total, 4000 to 5000 households are selected for participation in the survey annually. All family members respond to the questionnaires individually. The response rate ranges from 78.4–81.9% each year^23^. As data collection was performed by highly skilled surveyors and controlled for quality, the data were considered highly accurate and reliable^23^. All KNHANES participants provided written consent to participate in the survey and consent to the use of their personal data. The KNHANES data are openly available in a public repository (https://knhanes.kdca.go.kr/knhanes/eng/index.do).

The current study used KNHANES (2007–2019) survey data, which included information on SHS, urine cotinine sampling, smoking history, and ADHD-related history in children and adolescents. Of the 105732 participants initially enrolled, we excluded those aged ≥19 years and those with missing data or refusal to provide data on age, sex, household income, self-rated health status, and smoking experience; and finally, 16434 participants were included. We divided the dataset into three analysis groups based on the KNHANES data design: Group 1 had 3212 participants with information on SHS exposure; Group 2 had participants with urine samples for urine cotinine levels; and Group 3 included participants with information on smoking history.

### Main variables

ADHD was confirmed in children and adolescents with physician-diagnosed ADHD who responded ‘yes’ to the healthcare interview question: ‘Have you ever been diagnosed with ADHD by a doctor?’. Children and adolescents with physician-diagnosed ADHD were also asked about their age at diagnosis using the following question: ‘If yes, how old were you at the time of the diagnosis?’.

The questionnaire contained questions on smoking experiences during their lifetime: ‘Have you ever smoked a cigarette?’ was used to define the smoking experience. The exposure to SHS at home during the day was defined with a ‘yes’ response to the following question: ‘Is there any person who smokes routinely inside the house?’. Participants were also asked about the number of smoking days and number of smoked cigarettes per day during the last month with the following questions: ‘In the past month, how many days did you smoke at least one cigarette?’ and ‘On average, how many cigarettes per day did you smoke in the past month?’. Smoking history was calculated using pack-days from the responses to the questions above.

Urine samples were collected from the selected participants by a trained pathologist using a urine specimen cup. The participants were requested to submit early morning samples whenever possible. All samples were stored in an icebox, maintained at 4–7°C, and transported to the authorized central laboratory. For the cotinine analysis, 175 μL diphenylamine, the internal standard, was added to the urine samples, then 1 mL of each urine sample was hydrolyzed with 50 μL of 0.1M sodium hydroxide and extracted with 500 μL of chloroform. After centrifugal separation (1900g, 10 min), the residue was dried using sodium sulfite. Urinary cotinine (ng/mL) was analyzed by gas chromatography–mass spectrometry using a PerkinElmer Clarus 600T instrument (PerkinElmer, Waltham, MA, USA)^24^. The German External Quality Assessment Scheme (G-EQUAS) was used to measure urine cotinine levels as part of external quality assurance and control.

### Covariables

Baseline characteristics, such as age, sex, and household income, were used in the current study. Household income was assessed in quartiles. Children and adolescents were asked to self-rate their health which was considered a confounding variable.

### Statistical analysis

Statistical analyses were conducted using the SAS statistical software (version 9.4; SAS Institute, Cary, NC, USA). ADHD status was calculated using the chi-squared test based on the baseline characteristics and smoking exposure status. A logistic regression analysis investigated the association between direct and indirect smoking exposure and ADHD. Direct smoking exposure was estimated based on smoking experience, and indirect smoking exposure was defined as SHS exposure. Crude and adjusted logistic regression models were used in this study. The logistic regression model was adjusted for age, sex, household income, and self-rated health status. The adjusting factors were selected in a stepwise backward manner. Linear regression models were developed to demonstrate the dose-response relationship between direct/indirect exposure to smoking and age at ADHD diagnosis. Direct smoking exposure was estimated by smoking history calculated by pack-days in the last month, while indirect smoking exposure was defined as the urine cotinine level, a continuous variable. Adjusted linear regression models were developed after adjusting for age, sex, household income, and self-rated health status; a two-tailed p<0.05 was considered statistically significant for all statistical calculations.

## RESULTS

**Figure 1:**
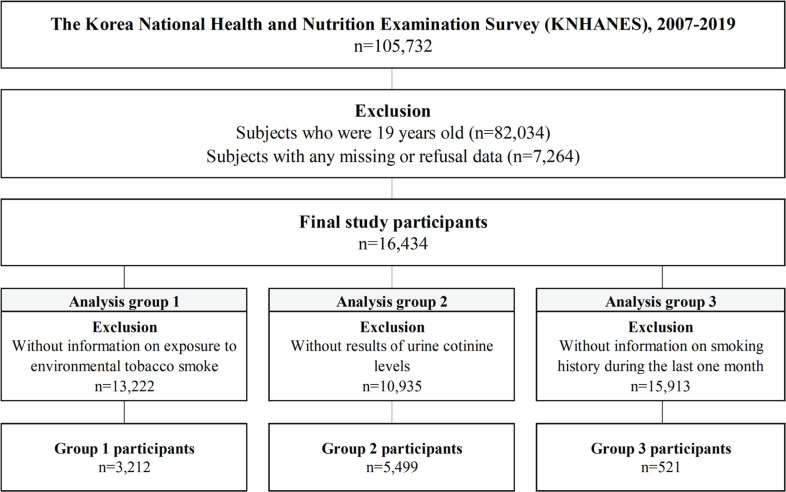
Schematic diagram depicting study participants

We retrieved data from 105732 participants of the 2007–2016 KNHANES and excluded 89298 participants because they were aged ≥19 years and had missing or refused to provide data. Therefore, 16434 participants aged <19 years were included in this analysis and divided into three groups. Analysis Group 1 included 3212 participants with data on SHS, analysis Group 2 had 5499 participants with data on urine cotinine levels, and analysis Group 3 included 521 participants with a smoking history within the last month (Figure 1).

Of the total participants, 133 were diagnosed with ADHD (0.8%), 58 (43.6%) were aged <12 years, and 75 (56.3%) were aged ≥12 years. A greater proportion of the children with ADHD were males (M:F=99:34), and the second quartile of household income was more prominently distributed in the non-ADHD and ADHD groups. In all, 1081 participants reported a smoking history, and 16 had ADHD. In the analysis Group 1, 1672 participants answered that they were exposed to SHS, including 14 children with ADHD. In analysis Group 2, the group with ADHD (n=61, mean: 14.13 ng/mL) had higher urine cotinine levels than the group without ADHD (n=5438, mean: 9.96 ng/mL). In analysis Group 3, of the 521 participants with information on smoking history, the amount of smoking was higher in the ADHD group (17.33 pack-days) than in the non-ADHD group (8.18 packdays) (Table 1).

**Table 1:** Baseline characteristics of the study participants (children and adolescents) according to the attention deficit hyperactivity disorder (ADHD), KHANES (2007–2019) (N=16434)

	*ADHD n (%)*		*p*
	*No*	*Yes*	
**Total**	16301 (99.2)	133 (0.8)	
**Age** (years)			<0.05
<12	10336 (99.4)	58 (0.6)	
<19	5965 (98.8)	75 (1.2)	
**Sex**			<0.05
Male	8570 (98.9)	99 (1.1)	
Female	7731 (99.6)	34 (0.4)	
**Household income quartile**			0.290
1st	1476 (99.1)	13 (0.9)	
2nd	4560 (99.0)	46 (1.0)	
3rd	5511 (99.3)	36 (0.7)	
4th	4754 (99.2)	38 (0.8)	
**Self-rated health**			<0.05
Good	11515 (99.4)	69 (0.6)	
Moderate	4141 (98.8)	50 (1.2)	
Bad	645 (97.9)	14 (2.1)	
**Smoking experience**			<0.05
No	15220 (99.2)	117 (0.8)	
Yes	1081 (98.5)	16 (1.5)	
Analysis Group 1*			
Total	3179 (99.0)	33 (1.0)	
**Environmental tobacco smoke**			<0.05
Non-exposed	2507 (99.2)	19 (0.8)	
Exposed	672 (98.0)	14 (2.0)	
Analysis Group 2**			
Total	5438 (98.9)	61 (1.1)	
Urine cotinine level, mean ± SE	9.96 ± 0.59	14.13 ± 7.52	<0.05
Analysis Group 3***			
Total	513 (98.5)	8 (1.5)	
Smoking history (pack-days), mean ± SE	8.18 ± 0.46	17.33 ± 7.96	<0.05

* Analysis Group 1 included subjects with information on exposure to environmental tobacco smoke (n=3212).

** Analysis Group 2 included subjects with results of urine cotinine level (n=5499).

*** Analysis Group 3 included subjects with information on smoking history during the last one month (n=521). SE: standard error.

The results of the logistic regression model showed that smoking experience was significantly associated with ADHD (OR=1.48; 95% CI: 1.14–3.26; and AOR=1.22; 95% CI: 1.02–1.64). However, the association between SHS exposure and ADHD was attenuated after the adjustment (OR=2.42; 95% CI: 1.08–4.02; and AOR=1.42; 95% CI: 0.86–2.64) (Table 2).

**Table 2:** The risk of the attention deficit hyperactivity disorder (ADHD) from the logistic regression model based on smoking experience and environmental tobacco smoke, KHANES (2007–2019)

	*OR (95% CI)*	*AOR (95% CI)*
**Smoking experience**		
No (Ref.)		1
Yes	1.93 (1.14–3.26)	1.22 (1.02–1.64)
**Environmental tobacco smoke**		
Non-exposed (Ref.)		1
Exposed	2.42 (1.08–4.02)	1.42 (0.86–2.64)

AOR: adjusted odds ratio; adjusted for age, sex, household income, and self-rated health status.

Linear regression models based on the age at diagnosis of ADHD and smoking history or urine cotinine level, smoking history, and urine cotinine level were statistically correlated with the age at ADHD diagnosis. The higher the number of cigarettes smoked, the younger the age at ADHD diagnosis (crude β= -1.80; adjusted β= -1.52) (Table 3).

**Table 3:** Results of linear regression models on age of diagnosis of the attention deficit hyperactivity disorder (ADHD) and smoking history or urine cotinine level, KHANES (2007–2019)

	*Diagnosed age of ADHD*			
	*Crude β (SE)*	*R^2^*	*Adjusted β (SE)*	*R^2^*
Smoking history (pack-days)	**-1.8038 (0.6765)**	0.3108	**-1.5254 (0.6617)**	0.1101
Urine cotinine level (ng/mL)	**-0.0074 (0.0119)**	0.0541	-0.0069 (0.0114)	0.0104

The adjusted model had adjustments for age, sex, household income, and self-rated health status. Bold values indicate statistically significant relationships.

## DISCUSSION

The current study investigated the association between ADHD, smoking experience, and SHS exposure among Korean children and adolescents and found that smoking exposure was closely associated with ADHD. The age at ADHD diagnosis was significantly younger among adolescents with a higher smoking experience than among others. SHS exposure and urinary cotinine levels were related to ADHD; however, these were attenuated after adjustment.

Clinically, smoking is closely related to ADHD in young people who had ADHD. Adolescents who tended to smoke more had increased symptoms of ADHD, such as inattention or hyperactivity^25,26^. The progression of ADHD was quickly aggravated in young patients with ADHD with smoking experience, and cravings were much more severe while quitting smoking^27,28^. A 16-year longitudinal study reported that the group with ADHD had higher rates of daily smoking and more severe withdrawal symptoms than the non-ADHD children and adolescent groups^27^.

Several mechanisms are responsible for how smoking results in ADHD. The suggested theory of ADHD pathophysiology is associated with dopamine dysfunction, which leads to disrupted reinforcement resulting in executive dysfunction^1,29^. Animal models in which the dopamine transporter gene was knocked down or knocked out exhibited an ADHDlike phenotype, which increases activity levels that normalize to wild-type levels with stimulant administration^22^. Smoking also increases dopamine production, associated with dopamine release, through accelerated atrioventricular conduction^30^. The nicotine-dependent human brain is also involved in neuronal processing-mediated working memory^31^. Smoking, which shares a similar mechanism with ADHD, results in adverse outcomes on human traits of executive function.

We also found that SHS exposure was associated with a higher risk of ADHD, even though the statistical significance was attenuated after adjustment. SHS exposure and infant and young children’s health are associated with increased respiratory tract infections, neurocognitive problems, and adolescent smoking^32^. Zubair et al.^33^ reported that children exposed to SHS at home had significantly increased odds of having more than two neurobehavioral disorders, including ADHD, learning disabilities, and conduct disorders, compared to those not exposed to SHS^20^. A teacher-rated rating scale in Korea confirmed that SHS exposure in children is associated with neurocognitive dysfunction such as inattentiveness and hyperactive performance^21^. SHS exposure increases executive function problems in rodent models^33^. A large population-based study reported that paternal smoking during pregnancy increased the risk of ADHD^34^. Our data support the hypothesis that the association between SHS exposure and ADHD is statistically significant, with a need for follow-up studies.

Our study showed that the amount of smoking is related to the diagnosis of ADHD at an early age. Thus, age and nicotine exposure may influence executive brain function^35^. Brain maturation is not completed before puberty, and human prefrontal cortex conducting executive function reaches stability at approximately 12 years of age^36^. Early exposure to nicotine interferes with cognitive development and leads to an executive deficit^35,37^. Young people with a previous ADHD history tend to start smoking early^38^. Moreover, ADHD is difficult to diagnose at an undisrupted daily life stage. Exposure to smoking in preschool children before ADHD diagnosis may increase the risk of ADHD expression compared with non-smokers^39^.

Although the statistical significance was attenuated after adjustment, our results showed that urine cotinine levels were also associated with the risk of ADHD and the age at ADHD diagnosis. In an experimental mouse model, exposure to cigarette aerosols during the early life stage (detected through urine cotinine levels) altered frontal cortex development^40^. Young smokers may have a riskier decision than non-smokers because of immature inhibitory control^41^. Additionally, youths exposed to smoking from family members, siblings, and relatives may be at a greater risk of developing a nicotine habit than those without smoking exposure^42,43^. Voluntary smoke-free home policies can reduce the environmental exposure range in children by 20–50%^44^. Therefore, the smoking history of family members may influence potentially causing ADHD in children and adolescents, necessitating education and counselling on smoking cessation and abstinence in their families.

### Strengths and limitations

This study has several strengths. To our knowledge, this is the first study showing the relationship between ADHD and smoking experience with detailed information on smoking, such as urine cotinine levels, period of smoking, and SHS exposure, using data from 2007 to 2019 of the KNHANES survey which was reliably designed and conducted as a governmentled nationwide survey with elaborately sampled participants.

However, the study design may have limited the findings of this study. The KNHANES was a crosssectional study, and the interpretation of the causal effect of smoking on ADHD was limited. Nevertheless, we conducted several analyses using different definitions of tobacco exposure. We demonstrated an association between SHS exposure and ADHD or urine cotinine levels and age at ADHD diagnosis, even with an attenuated association after adjusting for a limited number of participants. The KNHANES collected data based on questionnaires, omitting confounders, such as genetic factors. In addition, the laboratory results showed a short exposure period because urine cotinine has a short half-life, and the smoking history only described events during the last month. Thus, further longitudinal studies with detailed information are needed to establish a relationship between ADHD and smoking.

## CONCLUSIONS

The results of this study demonstrated an association between smoking and ADHD. Smoking status and high and indirect exposure among children and adolescents are associated with ADHD. Additional studies on the prevention of smoking exposure in infants and young children are warranted.

## Data Availability

The data supporting this research are available from the following source: https://knhanes.kdca.go.kr/knhanes/eng/index.do
